# Dyrk1 inhibition improves Alzheimer's disease‐like pathology

**DOI:** 10.1111/acel.12648

**Published:** 2017-08-04

**Authors:** Caterina Branca, Darren M. Shaw, Ramona Belfiore, Vijay Gokhale, Arthur Y. Shaw, Christopher Foley, Breland Smith, Christopher Hulme, Travis Dunckley, Bessie Meechoovet, Antonella Caccamo, Salvatore Oddo

**Affiliations:** ^1^ The Arizona State University‐Banner Neurodegenerative Disease Research Center at the Biodesign Institute Arizona State University Tempe AZ 85287 USA; ^2^ Department of Biomedical and Biotechnological Sciences University of Catania Catania 95125 Italy; ^3^ Division of Drug Discovery and Development Department of Pharmacology and Toxicology College of Pharmacy The University of Arizona Tucson AZ 85721 USA; ^4^ Department of Chemistry & Biochemistry The University of Arizona Tucson AZ 85721 USA; ^5^ Neurogenomics Division Translational Genomics Research Institute Phoenix AZ 85004 USA; ^6^ School of Life Sciences Arizona State University Tempe AZ 85287 USA

**Keywords:** AD, Alzheimer's disease, amyloid beta, plaques, tangles, 3xTg‐AD

## Abstract

There is an urgent need for the development of new therapeutic strategies for Alzheimer's disease (AD). The dual‐specificity tyrosine phosphorylation‐regulated kinase‐1A (Dyrk1a) is a protein kinase that phosphorylates the amyloid precursor protein (APP) and tau and thus represents a link between two key proteins involved in AD pathogenesis. Furthermore, Dyrk1a is upregulated in postmortem human brains, and high levels of Dyrk1a are associated with mental retardation. Here, we sought to determine the effects of Dyrk1 inhibition on AD‐like pathology developed by 3xTg‐AD mice, a widely used animal model of AD. We dosed 10‐month‐old 3xTg‐AD and nontransgenic (NonTg) mice with a Dyrk1 inhibitor (Dyrk1‐inh) or vehicle for eight weeks. During the last three weeks of treatment, we tested the mice in a battery of behavioral tests. The brains were then analyzed for the pathological markers of AD. We found that chronic Dyrk1 inhibition reversed cognitive deficits in 3xTg‐AD mice. These effects were associated with a reduction in amyloid‐β (Aβ) and tau pathology. Mechanistically, Dyrk1 inhibition reduced APP and insoluble tau phosphorylation. The reduction in APP phosphorylation increased its turnover and decreased Aβ levels. These results suggest that targeting Dyrk1 could represent a new viable therapeutic approach for AD.

## Introduction

Alzheimer's disease (AD) is the most common neurodegenerative disorder, which affects about 5.5 million people in the United States and 40 million worldwide (Alzheimer's, 2015). There is an urgent need for developing new therapeutic approaches as the current FDA approved medications have no effects on the progression of the disease. If the current *status quo* is not altered by the introduction of new therapeutic strategies able to slow down or halt the progression of the disease, it is estimated that by 2050, 12 million people in the United States will have AD (Alzheimer's, 2015).

Accumulation of amyloid‐β (Aβ) and hyperphosphorylated tau is a critical event in the pathogenesis of AD (Querfurth & LaFerla, 2010). Tau is a microtubule‐binding protein; one of the most well‐known functions of tau is to bind to and stabilize microtubules (Querfurth & LaFerla, 2010). This property is regulated by phosphorylation events, with phosphorylated tau having less affinity for microtubules. Pathological tau is hyperphosphorylated and produces soluble and insoluble inclusions, which form neurofibrillary tangles (NFTs) characteristic of AD and other tauopathies (Medina *et al*., 2016). To this end, inhibiting the activity of known tau kinases could be a valid approach to reduce the formation of NFTs (Rojas & Boxer, 2016). Aβ is the major component of extracellular plaques that accumulate throughout the brain of people with AD (Querfurth & LaFerla, 2010). Aβ is made from a larger precursor, known as amyloid precursor protein (APP). APP is sequentially cleaved by BACE1 and the γ‐secretase complex to generate Aβ (Medina *et al*., 2016). Full‐length APP undergoes several post‐translational modifications, including phosphorylation at specific epitopes that regulates its half‐life and affinity for BACE1 (Lee *et al*., 2003; Ma *et al*., 2012). This is particularly intriguing as reducing APP levels and/or reducing its affinity of BACE1 could be valid approaches to lower Aβ levels.

The dual‐specificity tyrosine phosphorylation‐regulated kinase‐1A (Dyrk1a) is a protein kinase member of the Dyrk family, which includes five kinases (Duchon & Herault, 2016). The DYRK1a gene is located in the Down syndrome critical region on chromosome 21, which results in a 1.5‐fold increase of Dyrk1a protein levels (Tejedor & Hammerle, 2011). Dyrk1a levels are tightly regulated, as both overexpression and lack of Dyrk1a activity are associated with mental retardation (Luco *et al*., 2016). Dyrk1a can directly phosphorylate tau on numerous serine and threonine residues (Ryoo *et al*., 2007; Azorsa *et al*., 2010). To this end, overexpression of Dyrk1a contributes to the accumulation of NFT in Down syndrome (Liu *et al*., 2008). In addition to its effects on tau pathology, Dyrk1a appears to be linked to APP/Aβ metabolism. For example, in primary rat cortical neurons, Dyrk1a inhibition reduces tau phosphorylation at multiple epitopes in a dose‐dependent manner (Coutadeur *et al*., 2015). The same inhibitor reduced Aβ production in HEK293 cells overexpressing APP (Coutadeur *et al*., 2015). This is consistent with earlier findings showing that Dyrk1a phosphorylates APP and enhances its affinity for BACE1 and the γ‐secretase complex, thereby increasing overall Aβ levels and plaque deposition (Wegiel *et al*., 2011). Early evidence also suggests that Aβ accumulation can facilitate Dyrk1a activity (Kimura *et al*., 2007), creating a vicious cycle. Consistent with these observations, Dyrk1a levels are increased in postmortem human AD brains (Ferrer *et al*., 2005). Together, these data suggest that reducing Dyrk1a activity could be a valid therapeutic strategy for targeting both Aβ and tau in AD (Adayev *et al*., 2011; Smith *et al*., 2012). In this study, we test the effects of a new Dyrk1 inhibitor on the AD‐like phenotype of 3xTg‐AD mice, a widely used animal model of AD (Oddo *et al*., 2003).

## Results

### Pharmacological characteristics of Dyrk‐inh

The objective of this study was to assess whether Dyrk1 is a valid therapeutic target for AD. To this end, we utilized a newly synthesized Dyrk1 inhibitor (Dyrk1‐inh). Dyrk1‐inh is a benzimidazole‐like compound with a molecular weight in the 310‐350 range (Fig. S1A; the synthesis of Dyrk1‐inh is shown in Patent number US2016/050198). Dyrk1‐inh is an ATP‐competitive kinase inhibitor with excellent intrinsic affinity and a clear dose‐dependent inhibitory effect on Dyrk1a activity (IC_50_: 34 nm; Fig. S2A). To probe for selectivity, we analyzed the inhibitory effect of 10 μm Dyrk1‐inh using the EZ reader electrophoresis mobility chip instrument (see Methods). The test was ran against different kinases known to be involved in neurodegenerative processes, such as GSK3β, CDK5, and CK1δ. We found that at this concentration, Dyrk1‐inh inhibited >99% of Dyrk1a and Dyrk1b activity (IC_50_: 124 and 129 nm, respectively; Fig. S2B). Dyrk1‐inh also had a modest effect on the activity of GSK3β and CDK5 (Fig. S2B). To evaluate the permeability of Dyrk1‐inh across the blood‐brain barrier (BBB), we utilized standard surrogate BBB assays such as the Parallel Artificial Membrane Permeability Assay and Caco‐2. We found that Dyrk1‐inh had a value of effective permeability (P_e_) of 26.50 E‐06 cm s^−1^, which indicated that passive diffusion across the BBB was likely. Using the Caco‐2 permeability test, we found that Dyrk1‐inh had an efflux ratio of 0.5 (diffusion in an apical‐to‐basolateral direction = 5.71; diffusion in a basolateral‐to‐apical direction = 3.13) through a monolayer of Caco‐2 cells. Taken together, these results suggest that Dyrk1‐inh crosses the BBB with no significant efflux liabilities.

### Dyrk1 inhibition improves learning and memory

To determine the effects of chronic inhibition of Dyrk1 on AD‐like pathology, we treated 10‐month‐old female 3xTg‐AD (*n *= 13) and NonTg (*n *= 15) mice with a Dyrk1 inhibitor (Dyrk1‐inh), which was delivered via daily intraperitoneal (i.p.) injections for eight weeks (12.5 mg kg^−1^). Herein, we refer to these mice as 3xTg‐AD/DYR and NonTg/DYR. Age‐ and gender‐matched 3xTg‐AD (*n *= 12) and NonTg (*n *= 13) mice were injected with vehicle and used as control groups. Herein, we refer to these mice as 3xTg‐AD/veh and NonTg/veh (Fig. S3A). During the dosing period, we weighed mice weekly and found that the body weight of mice in the 3xTg‐AD/veh group was significantly higher than all the other groups (Fig. S3B). At this age, 3xTg‐AD mice are overweight, and the treatment with Dyrk1‐inh restored the normal body weight. Notably, no effect for the treatment was elicited in NonTg mice (Fig. S3B).

**Figure 1:**
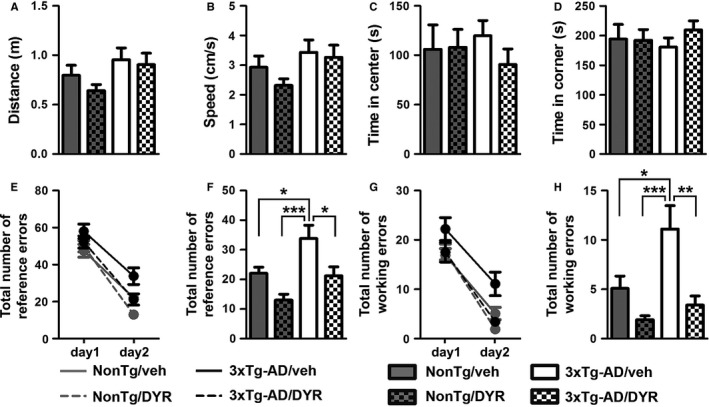
Chronic Dyrk1 inhibition improves learning and memory in 3xTg‐AD mice. (A, B) The graphs show total distance traveled and speed during the open‐field test. The data were not statistically significant among the four groups (*P *=* *0.0957 and 0.1016, respectively). (C, D) The graphs show the time spent in the center and periphery of the arena during open‐field testing. The data were not statistically significant among the four groups (*P *=* *0.7828 and 0.7827, respectively). (E‐H) We then evaluated the performance of the mice in the radial arm water maze (RAWM) by scoring spatial and working memory errors. The graphs show the average of the total errors that each mouse made across the 15 trials/day. (E) All groups show a decrease in total spatial errors at day 2, indicating learning (time effect, *P *<* *0.0001; group effect, *P *=* *0.0002; interaction effect, *P *=* *0.1841). (F) When we analyzed the total number of spatial errors at day 2, we found significant differences between groups (*P *=* *0.0001). As indicated in the figure, post hoc analyses showed that 3xTg‐AD/veh mice performed worse than all the other groups. Notably, 3xTg‐AD/DYR mice performed better than 3xTg‐AD/veh mice (*P *<* *0.05) and as well as NonTg mice (*P *>* *0.05). (G, H) Working memory errors: All the groups learned the task (time effect, *P *<* *0.0001; group effect, *P *=* *0.0001 interaction effect, *P *=* *0.4716). However, on day 2, we found significant differences between groups (*P *<* *0.0001). Post hoc analyses indicated that 3xTg‐AD/DYR mice performed significantly better than 3xTg‐AD/veh mice (*P *<* *0.01) and as well as NonTg mice (*P *>* *0.05). Both for working and reference errors, no differences were detected between NonTg/veh and NonTg/DYR mice (*P *>* *0.05). Data are presented as means ± SEM. Data in panels A‐D, F, and H were analyzed by one‐way ANOVA followed by Tukey's post hoc analyses. Data in panels E and G were analyzed by two‐way ANOVA. *P < 0.05; **P < 0.01; ***P < 0.001.

During the last 3 weeks of i.p. injections, mice were tested in a series of cognitive and noncognitive behavioral tests and were sacrificed immediately after the completion of the behavioral tests (Fig. S3A). We first used the open‐field activity test to measure general motor function. We found that spontaneous activity and gross motor function were similar among the four groups (Fig. 1A–B), as indicated by the total distance traveled and the average speed in the activity chamber during the test. To evaluate general anxiety and stress, we measured open‐field thigmotaxis and the time spent in the center of the activity chamber. We found that the time spent in the periphery and the center was similar among the four groups (Fig. 1C–D). Taken together, these data indicate that Dyrk1 inhibition has no effects on body weight, general motor function, and anxiety‐like behavior, suggesting that under these experimental conditions, the drug was well tolerated.

To assess the effect of the treatment on cognitive function, we tested mice in the radial arm water maze. This task is routinely used to measure reference and working memory, two memory domains mainly dependent on the hippocampus and frontal cortex, respectively (Hodges, 1996). During the first day of training, mice received 15 consecutive trials to find a platform, which is alternated between visible and hidden platform. Twenty‐four hours after the last training trial, mice received 15 additional trials, during which the platform was always hidden. The total entries in arms without the platform were considered reference memory errors, while the number of reentries in an arm without the platform during the same trial was considered as working memory errors. We found that the overall reference memory errors between day 1 and day 2 were significantly reduced for all four groups (Fig. 1E, *P *< 0.0001), which suggests that all groups learned the task. In contrast, we found that during day 2, there were significant differences among groups (one‐way ANOVA, *P* = 0.0001). Post hoc analyses with Tukey's correction showed that 3xTg‐AD/veh mice made significantly more errors compared to all the other groups (Fig. 1F). Notably, the treatment mitigated these deficits; indeed, 3xTg‐AD/DYR performed significantly better than 3xTg‐AD/veh and NonTg/veh mice (Fig. 1F). We found similar results when we measured working memory errors. To this end, 3xTg‐AD/veh mice performed worse compared to all the other groups. More importantly, 3xTg‐AD/DYR mice performed significantly better than 3xTg‐AD/veh mice (*P* < 0.01) and as well as NonTg/veh mice (Fig. 1G–H; *P *>* *0.05). Taken together, these data indicate that Dyrk1 inhibition significantly improves cognitive deficits in 3xTg‐AD mice.

### Dyrk1 inhibition decreases insoluble tau phosphorylation

**Figure 2:**
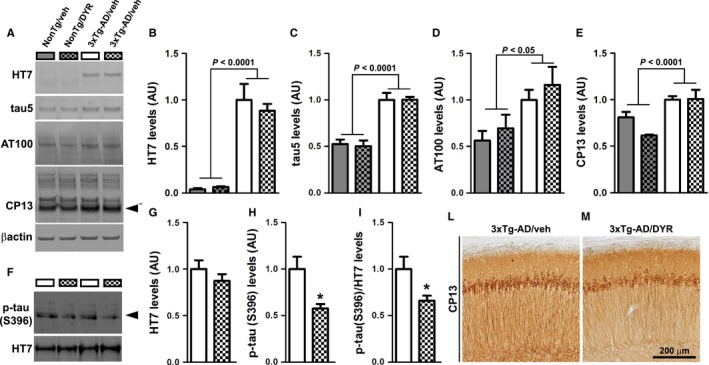
Chronic Dyrk1 inhibition reduces tau pathology. (A) Western blot on brain lysates probed with the indicated antibodies (*n *= 4 mice/group for NonTg and *n *= 5 mice/group for 3xTg‐AD). (B) Quantitative analysis of the HT7 blot showed a genotype effect (*P *<* *0.0001), as expected, but neither a treatment nor an interaction effect (*P *=* *0.5452 and 0.4102, respectively). (C) Quantitative analysis of the tau5 blot showed a genotype effect (*P *<* *0.0001), but neither a treatment nor an interaction effect (*P *=* *0.9146 and 0.8895, respectively). (D) Quantitative analysis of the AT100 blot showed a genotype effect (*P* = 0.0117), but neither a treatment nor an interaction effect (*P* = 0.4482 and 0.7140, respectively). (E) Quantitative analysis of the steady‐state levels of ~50 kDa band of CP13 (black arrowhead) showed a genotype effect (*P *<* *0.0001), but neither a treatment nor an interaction effect (*P *=* *0.2223 and 0.0662, respectively). (F–I) Western blot and quantitative analyses on insoluble brain fraction probed for total and p‐tau (S396). Quantitative analyses of the blots showed that total tau levels were similar between the two groups (*P *=* *0.2890). In contrast, the steady‐state levels of ~50 kDa band (black arrowhead) normalized to protein concentration or to total tau levels were significantly decreased in the 3xTg‐AD/DYR compared to 3xTg‐AD/veh (*P *=* *0.005 and 0.0211, respectively). (L‐M) Representative photomicrographs of treated and untreated 3xTg‐AD brain slices probed with the CP13 antibody (*n *= 6 mice/group). Data in panels B‐E were normalized to β‐actin, presented as means ± SEM, and analyzed by two‐way ANOVA (genotype/treatment). Data in panels G‐H were normalized to protein concentration, presented as means ± SEM, and analyzed by Student's t‐test. *P < 0.05.

To identify the neuropathological correlates of the improved cognitive function in 3xTg‐AD/DYR mice, at the end of the behavioral tests, we analyzed their brains using biochemical and histological approaches. Initially, we measured tau levels by Western blot. Specifically, we analyzed human and endogenous soluble tau levels using the HT7 and tau5 antibodies, respectively. Consistent with the presence of the tau transgene, both 3xTg‐AD groups had significantly higher HT7 levels compared to both NonTg groups (*P *<* *0.0001). However, Dyrk1‐inh had no effect on HT7 levels as we found no statistically significant difference between 3xTg‐AD/veh and 3xTg‐AD/DYR mice (Fig. 2A–B). These data suggest that Dyrk1‐inh does not alter the expression of the transgene. We found similar results when we measured endogenous mouse tau levels: both 3xTg‐AD groups had significantly higher tau5 levels than the two NonTg groups (*P *<* *0.0001), while the treatment did not alter total tau levels (Fig. 2A,C). To further measure the effects on tau phosphorylation, we used CP13, an antibody raised against tau phosphorylated at Ser202. We found that 3xTg‐AD mice had higher CP13 levels than NonTg mice (*P *<* *0.0001), which were not affected by the treatment (Fig. 2A,D). Given that hyperphosphorylated tau forms insoluble aggregates, we measured the levels of insoluble total and phosphorylated tau (S396). While the insoluble levels of total tau, as detected by the HT7 antibody, were similar between treated and untreated 3xTg‐AD mice (Fig. 2F–G), we found that Dyrk1‐inh significantly reduced the levels of insoluble tau phosphorylated at S396 in 3xTg‐AD mice (Fig. 2F,H–I; *P* = 0.005 and 0.021, respectively). Consistent with the Western blot data, immunostaining of hippocampal sections from 3xTg‐AD/veh and 3xTg‐AD/DYR mice with the CP13 antibody showed similar immunoreactivity between the two groups (Fig. 2L–M). Together these data indicate that Dyrk1‐inh selectively reduces insoluble tau phosphorylation. Further studies are needed to identify the mechanisms underlying this selectivity.

### Dyrk1 inhibition reduces Aβ pathology

**Figure 3:**
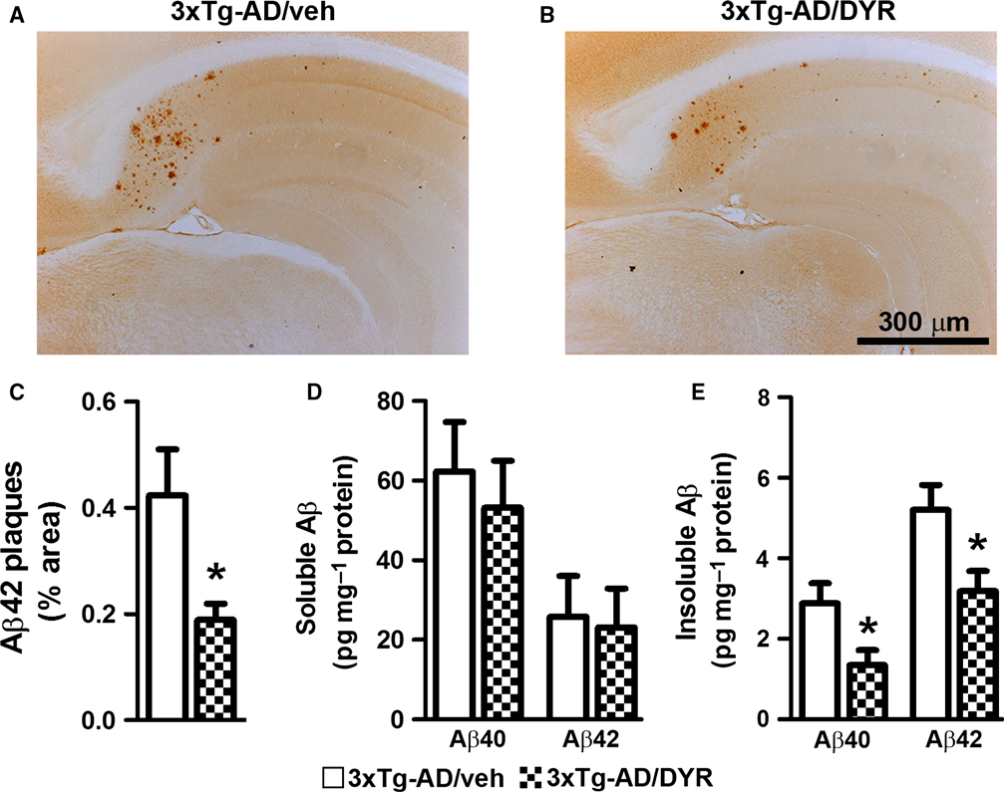
Dyrk1 inhibition reduces amyloid‐β (Aβ) pathology. (A‐B) Representative photomicrographs of 3xTg‐AD mice treated with Dyrk1‐inh or vehicle (*n *= 6 mice/group). Sections were immunostained with an Aβ42‐specific antibody from Millipore. (C) The graph shows a significant decrease in the average area occupied in the hippocampus by plaques in treated vs. untreated 3xTg‐AD mice (*P *=* *0.0218). (D) Enzyme‐linked immunosorbent assay measurements from brain lysates (*n *= 11 mice/group) revealed no difference in both Aβ40 and Aβ42 levels in the soluble fraction (*P *=* *0.6010 and *P* = 0.8539, respectively). (E) In contrast, insoluble Aβ40 and Aβ42 levels were significantly reduced in the brains of 3xTg‐AD/DYR mice compared to 3xTg‐AD/veh (*P *=* *0.0215 and *P *=* *0.0239, respectively). Data are presented as means ± SEM and were analyzed by Student's *t‐*test. *P < 0.05.

In addition to tau pathology, 3xTg‐AD mice show age‐dependent accumulation of Aβ pathology (Oddo *et al*., 2003, 2008). To determine the effects of Dyrk1‐inh on Aβ load, we immunostained hippocampal sections from 3xTg‐AD/DYR and 3xTg‐AD/veh mice with an Aβ42 specific antibody. We found that Aβ immunoreactivity was markedly reduced in 3xTg‐AD/DYR mice compared to 3xTg‐AD/veh mice (Fig. 3A–B). Quantitative analysis of the plaque load showed that this difference was statistically significant (Fig. 3C; *P *=* *0.0218). To further assess the effects of Dyrk1‐inh on Aβ pathology, we measured Aβ levels by sandwich ELISA. Surprisingly, we found that soluble Aβ40 and Aβ42 levels were not statistically different between 3xTg‐AD/DYR and 3xTg‐AD/veh mice (Fig. 3D). In contrast, insoluble Aβ40 and Aβ42 levels were significantly lower in 3xTg‐AD/DYR mice compared to 3xTg‐AD/veh mice (Fig. 3E, *P* = 0.0215 and 0.0239, respectively), which is consistent with the effects on Aβ load measured by immunohistochemistry.

**Figure 4:**
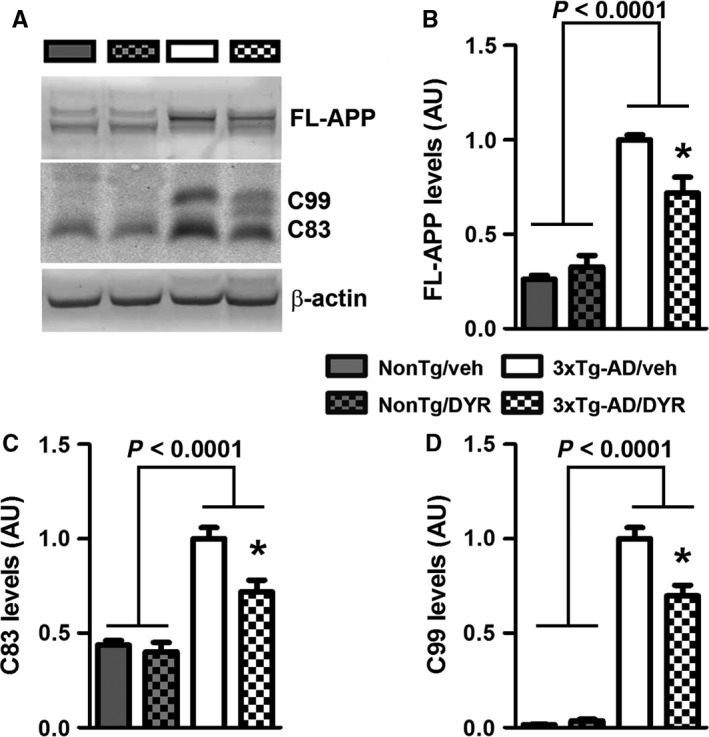
Dyrk1 inhibition alters APP processing. (A) Representative Western blots of proteins extracted from the brains of treated and untreated NonTg (*n *= 4 mice/group) and 3xTg‐AD (*n *= 5 mice/group) mice. Blots were probed with the indicated antibodies. (B) Quantitative analysis of the full‐length APP blot showed a genotype effect (*P *<* *0.0001), and a genotype‐treatment interaction effect (*P *=* *0.0074). Moreover, Bonferroni's post hoc analysis showed a significant reduction of full‐length APP for 3xTg‐AD/DYR (*P* < 0.01). (C–D) Quantitative analysis of the C83 and C99 blots showed a genotype effect (*P *<* *0.0001). Moreover, C99 and C83 levels were significantly decreased by treatment (*P *<* *0.01). Data were normalized to β‐actin, presented as means ± SEM, and analyzed by two‐way ANOVA (genotype/treatment) followed by *post hoc* Bonferroni's comparison. *P < 0.05.

**Figure 5:**
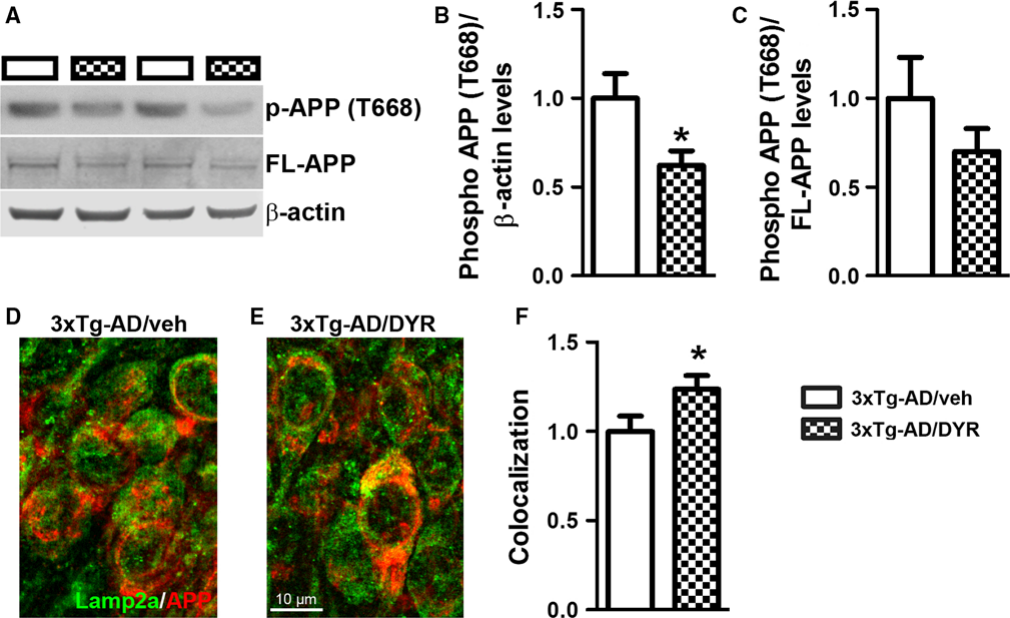
Dyrk1 inhibition reduces APP phosphorylation, thereby modifying APP turnover. (A) Representative Western blots of proteins extracted from the hippocampi of treated and untreated 3xTg‐AD mice (*n *= 9 mice/group). Blots were probed with the indicated antibodies. (B‐C) Quantitative analysis of the blots showed that while Dyrk1 inhibition did not change the ratio of phosphorylated over total APP, it significantly reduced the overall steady‐state levels of phosphorylation of APP at Thr668 (*P *=* *0.0480). However, the ratio pAPP over total APP was not significantly different between the two groups. (D–E) Representative microphotographs of hippocampal sections immunostained with the indicated antibodies (*n *= 30 pictures from 6 mice/group). (F) Semiquantitative analysis showed that the number of yellow pixels (indicating a colocalization between APP and the lysosomal protein Lamp2A, analyzed as Pearson's correlation coefficient) was significantly higher in 3xTg‐AD/DYR mice compared with 3xTg‐AD/veh mice (*P* = 0.0436). Data are presented as means ± SEM and were analyzed by Student's *t*‐test. *P < 0.05.

**Figure 6:**
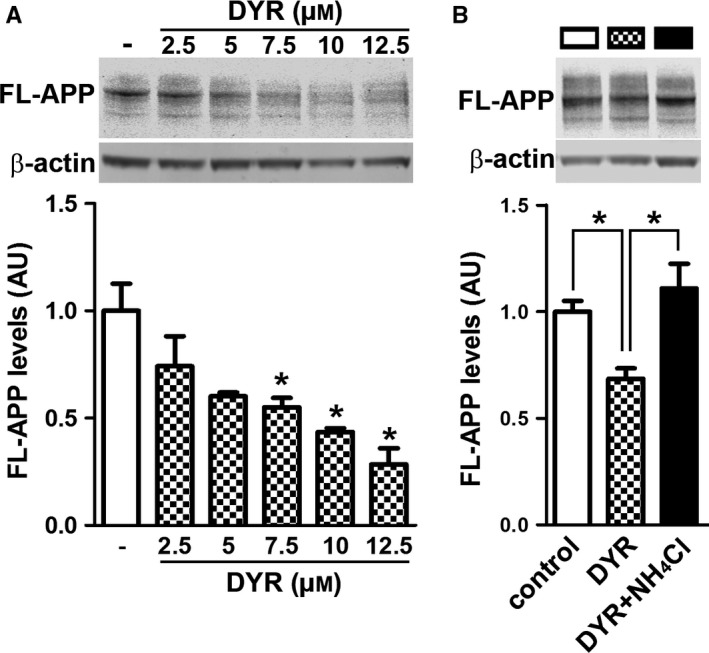
Dyrk1 inhibition reduces APP levels by a lysosomal‐dependent mechanism. (A) Immunoblot analysis (anti‐APP antibody clone 22C11) of total extracts from HT22 cells treated for 24 hours with different concentrations of Dyrk1‐inh. One‐way ANOVA analysis showed a significant effect (*P* = 0.0011). Post hoc analyses with Tukey's correction showed that the reduction in APP levels was significantly different starting at 7.5 μm Dyrk1‐inh. (B) Immunoblot analysis (anti‐APP antibody clone 22C11) of total extracts from HT22 cells treated for 24 h with 7.5 μm Dyrk1‐inh in the presence or absence of the lysosomal inhibitor ammonium chloride (2 mm). One‐way ANOVA analysis showed a significant effect (*P* = 0.0069). Post hoc analyses with Tukey's correction showed that the reduction in APP levels elicited by Dyrk1‐inh treatment is reversed by lysosomal inhibition. Data were generated by normalizing the levels of the protein of interest to β‐actin used as loading control. Results presented as means ± SEM of three independent experiments. *P < 0.05.

To begin understanding the mechanisms underlying the reduction in Aβ, we first focused on APP processing. We found that the 3xTg‐AD groups had higher full‐length APP levels compared to both NonTg groups (genotype effect, *P *<* *0.0001; Fig. 4A–B). Post hoc analyses indicated that APP levels were significantly lower in 3xTg‐AD/DYR mice compared to 3xTg‐AD/veh mice (*P *<* *0.01). Consistent with the APP levels, C83 and C99 levels were also subjected to a genotype effect (*P* < 0.0001). Specifically post hoc analyses revealed that C99 and C83 levels in 3xTg‐AD/DYR mice were significantly lower than 3xTg‐AD/veh mice (*P *<* *0.01; Fig. 4A,C–D). Taken together, these data indicate that the reduction in Aβ levels might be mediated by changes in APP levels and processing. To understand the mechanisms linking Dyrk1 inhibition to APP processing and Aβ production, we measured APP levels phosphorylated at Thr668 (APP/Thr668), which regulates APP turnover and processing (Lee *et al*., 2003; Ma *et al*., 2012). To this end, phosphorylation at Thr668 increases APP stability (Ma *et al*., 2012), enhancing BACE1 cleavage of APP. Further, APP/Thr668 levels are increased in AD human brains (Lee *et al*., 2003). We focused on APP/Thr668 as Dyrk1a is one of the known protein kinases responsible for phosphorylation of APP at this specific epitope (Ryoo *et al*., 2008). While the ratio of APP/Thr668 over total APP was not statistically significant between the two groups, we found that Dyrk1‐inh significantly reduces the overall steady‐state levels of APP/Thr668 levels (Fig. 5A–C; *P* = 0.048). To determine how the Dyrk1‐inh‐mediated changes in APP phosphorylation impact APP catabolism, we measured the amount of APP localized in the lysosomes by confocal microscopy. We found that the amount of colocalization between APP and the lysosomes was significantly increased in 3xTg‐AD/DYR mice compared to 3xTg‐AD/veh mice (Fig. 5C–E; *P* = 0.0436). To confirm a direct effect of Dyrk1 on APP levels and catabolism, we performed an *in vitro* experiment using the HT22 cell line. We treated cells for 24 h with increasing concentrations of Dyrk1‐inh or vehicle and found a decrease in APP levels in a dose‐dependent manner (Fig. 6A). To dissect the mechanisms of this reduction, using the same experimental condition, we inhibited the lysosome function (by adding ammonium chloride) and measured the levels of APP. Notably, inhibiting lysosomal function prevented the reduction in APP levels elicited by the Dyrk1‐inh (Fig. 6B). Overall, our data suggest that Dyrk1‐inh increased APP turnover, thus reducing Aβ production.

## Discussion

There are no current means to slow the progression of AD effectively. In this proof‐of‐concept study, we report that Dyrk1 is a valid new target for AD treatment and show that its chronic inhibition reduced Aβ and tau pathology and ameliorated cognitive deficits in 3xTg‐AD mice. These data are consistent with previous results indicating that CX‐4945, a selective and potent Dyrk1a inhibitor, reduces tau pathology in multiple systems (Kim *et al*., 2016). Taken together these results clearly indicate that Dyrk1 represents a valid therapeutic target for AD and support the development of new, effective, and clinically safe compounds aimed at reducing Dyrk1 activity.

In postmortem human AD patients, Dyrk1a activity is increased in several brain regions and is enriched in fractions containing phosphorylated tau, suggesting a possible contribution of this kinase in tau pathogenesis (Ferrer *et al*., 2005). The gene encoding Dyrk1a is located on chromosome 21, within the Down syndrome (DS) critical region (Ronan *et al*., 2009). Virtually 100% of people with DS will show brain accumulation of Aβ and tau in their fifth decade of life, and the vast majority of them will develop dementia by the age of 60 (Coppus *et al*., 2006; Head *et al*., 2012, 2016). To this end, several groups have suggested that Dyrk1a may represent a functional link between DS and AD (Kimura *et al*., 2007; Ryoo *et al*., 2007, 2008). For example, Dyrk1a phosphorylates tau at Thr212 *in vitro* (Woods *et al*., 2001), a residue that is hyperphosphorylated in AD. This is remarkable, as phosphorylation at this epitope primes tau for phosphorylation by GSK3β at other AD‐related epitopes (Woods *et al*., 2001). In mouse models, more contradictory results have been found. In DS mice, which are characterized by increased Dyrk1a activity, the levels of tau phosphorylated at several epitopes were increased (Liu *et al*., 2008). In contrast, transgenic mice overexpressing Dyrk1a do not show changes in tau phosphorylation at Thr212 (Ferrer *et al*., 2005). Together, these data suggest that the link between Dyrk1a and tau is complex. Our results indicate that Dyrk1 inhibition reduces tau phosphorylation only in the insoluble fraction; these findings are consistent with the observation that in human AD brains Dyrk1a is associated with tau in the sarkosyl‐insoluble fraction (Ferrer *et al*., 2005).

Dyrk1a has also been involved in APP processing and Aβ generation (Wegiel *et al*., 2011). Early work indicated that Dyrk1a, along with other kinases, directly phosphorylates APP at Thr668 *in vitro* (Park *et al*., 2007; Ryoo *et al*., 2008). Consistently, Dyrk1 transgenic mice show high levels of phosphorylated APP and increased Aβ levels (Ryoo *et al*., 2008). Furthermore, by phosphorylating APP and enhancing the amyloidogenic processing of APP, Dyrk1a increases Aβ production (Lee *et al*., 2003; Vingtdeux *et al*., 2005; Park *et al*., 2007). In postmortem human AD brains, the levels of APP phosphorylated at Thr668 are increased (Lee *et al*., 2003). Consistent with these observations, here we report that Dyrk1‐inh significantly decreases the steady‐state levels of APP phosphorylated at Thr668, which in turn leads to lower C99 levels and Aβ deposition.

In summary, we report that chronic Dyrk1 inhibition synergistically reduces Aβ and tau pathology. This is noteworthy as there is growing appreciation that combination therapies might be needed to successfully mitigate AD progression (Gao *et al*., 2016). Therefore, the identification of targets, that could affect different pathological markers, represents a step forward in developing new therapeutics for AD.

## Experimental procedures

### Mice

The 3xTg‐AD mice were generated as previously described (Oddo *et al*., 2003). In our colony of 3xTg‐AD mice, the AD‐like pathology in males is extremely variable. In contrast, the onset and progression of the phenotype in female 3xTg‐AD mice are very predictable, and the variability from animal to animal is minimal. Thus, only female mice were used. All mice were housed 4–5 per cage, kept on 12‐h light/dark cycle and were given *ad libitum* access to food and water. Animal care and treatments were in accordance with the applicable regulations in the vivarium (The Institutional Animal Care and Use Committee of the Banner Sun Health Research Institute).

### Dyrk1‐inh

Dyrk1‐inh was synthesized by Dr. Hulme at the University of Arizona. It is an ATP‐competitive kinase inhibitor with excellent intrinsic affinity and a clear dose‐dependent inhibitory effect on Dyrk1a activity (IC_50_: 34 nm; Fig. S1A). For the chronic treatment, Dyrk1‐inh was delivered via daily intraperitoneal (i.p.) injections for eight weeks, at 12.5 mg kg^−1^ in 50% PEG‐400 and 50% 0.9% NaCl. Control mice were injected with an equal volume of vehicle. Mouse weights were monitored throughout the dosing period.

### *In vitro* experiments

The Z’‐LYTE™ Detection Kinase Assay Kit (ThermoFisher Scientific, Waltham, MA, USA) was used to generate the inhibition curve. The Dyrk1a‐specific substrate peptide was labeled with a FRET pair (2 fluorophores, one at each end of the peptide). The reaction was conducted in the presence of recombinant Dyrk1a and 10 μm ATP. When a Dyrk1a inhibitor is present, the peptide is not phosphorylated, so it is more sensitive to cleavage. The peptide cleavage disrupts the FRET emission. By analyzing the emission ratio, it is possible to quantify the reaction progress. To evaluate selectivity, we then used the EZ Reader Electrophoresis Mobility Chip Instrument (Caliper Life Science, Hopkinton, MA, USA). This system allows the user to follow the reaction by analyzing the shift in electrophoretic mobility between the nonphosphorylated and phosphorylated peptides. The experiment was conducted according to the manufacturer's instructions in the presence of 45 μm ATP. The Parallel Artificial Membrane Permeability Assay (PAMPA) was performed using the BD Gentest^TM^ precoated PAMPA plate system (Analiza Inc, OH, USA), following the manufacturer's instructions. Caco‐2 cells (clone C2BBe1) were obtained from American Type Culture Collection (Manassas, VA, USA). Cell monolayers were grown to confluence on polycarbonate membranes in Costar Transwell^®^ plates. Cell monolayers were incubated at 37°C with 5% CO2 in a humidified incubator. Samples (in duplicate) were taken from the donor and receiver chambers at 120 min and were assayed by LC‐MS/MS using electrospray ionization. Further analysis of each monolayer was performed to ensure the integrity of the cell monolayers at the end of the experiment. The *in vitro* kinase assay used to evaluate Dyrk1‐inh capability of reducing tau phosphorylation at S396 was conducted as previously reported (Frost *et al*., 2011).

### Behavioral analyses

The open field is a commonly used qualitative and quantitative measure of general locomotor activity and willingness to explore in rodents. It was performed as previously described (Caccamo *et al*., 2012). Radial arm water maze. The radial arm water maze (RAWM) task is utilized to assess hippocampal‐dependent spatial reference and working memory, and it was performed as previously described (Medina *et al*., 2014). A video camera recorded each mouse. The experimenter, who was blind to the genotype and treatment, scored the entries into arms. The dependent measures were incorrect arm entries (reference memory errors) and reentries (working memory errors).

### Protein extraction

Mice were killed by CO_2_ asphyxiation and their brains were removed and sagittally bisected. Half of the brain was fixed in 4% paraformaldehyde and used for immunohistochemical experiments. The other half was collected and stored at −80°C until use. Frozen brains (without cerebellum) were processed as described previously (Caccamo *et al*., 2013). Briefly, brains were homogenized in a solution of tissue protein extraction reagent (T‐PER; ThermoFisher Scientific) containing 0.7 mg mL^−1^ of pepstatin A supplemented with a complete mini protease inhibitor tablet (Roche Applied Science, Indianapolis, IN, USA) and phosphatase inhibitors (Millipore, Billerica, MA, USA). The homogenized mixtures were centrifuged at 4°C for 1 h at 100 000 g, and the resulting supernatant was stored as the soluble fraction. The pellet was then homogenized in 70% formic acid and centrifuged as described above. The supernatant was stored as the insoluble fraction.

### Western blot and ELISA

Proteins from insoluble and soluble fractions were resolved by 10% Bis‐Tris SDS‐polyacrylamide gel electrophoresis (ThermoFisher Scientific) under reducing conditions and transferred to a nitrocellulose membrane. Membranes were developed as described previously (Orr *et al*., 2014).

Aβ40 and Aβ42 levels were determined with commercial ELISA kits (ThermoFisher Scientific), and experiments were conducted using the manufacturer's instructions.

### Immunohistochemistry and immunofluorescence

Brains were processed as previously described (Branca *et al*., 2014). Briefly, hemibrains were drop fixed in 4% paraformaldehyde in phosphate‐buffered saline for 48 h and then transferred into 0.02% sodium azide in phosphate‐buffered saline until slicing; 50‐μm‐thick free‐floating sections were subsequently obtained using a vibratome. For immunohistochemistry, sections were washed twice with TBS (100 mm Tris pH 7.4, 150 mm NaCl) and incubated for 30 min in 3% H_2_O_2_, to quench endogenous peroxidase activity. Next, sections were transferred into TBS‐A (100 mm Tris pH 7.4, 150 mm NaCl, 0.1% Triton X‐100) and TBS‐B (100 mm Tris pH 7.4, 150 mm NaCl, 0.1% Triton X‐100, 2% bovine serum albumin) for 15 and 30 min, respectively. Finally, the proper primary antibody was applied overnight at 4°C. Sections were washed to remove excess antibody and incubated in the suitable secondary antibody for 1 h at room temperature. Signal was enhanced by incubating sections in the avidin–biotin complex (Vector Labs) for 1 h. Sections were then washed and developed with diaminobenzidine substrate using the avidin–biotin horseradish peroxidase system (Vector Labs, Burlingame, CA, USA). Images were obtained with a digital Zeiss camera and analyzed using ImageJ. For immunofluorescence staining, the same protocol was followed with small differences. The step of quenching was skipped, and after the secondary antibody (AlexaFluor; ThermoFisher Scientific), the slices were mounted and coverslip with Prolong^®^ diamond mounting (ThermoFisher Scientific). Images were obtained with a Leica Confocal microscope and analyzed using ImageJ. For all colocalization measurements, lasers 561 nm and 488 nm were used for excitation of secondary antibody fluorophores Alexa555 and Alexa488, respectively. To obtain a Pearson correlation coefficient (PCC), ImageJ plugin ‘Coloc2’ was used. To quantify Aβ pathology, images from six mice/group were taken with a Zeiss AxioImager A1 using a 5× objective. Images were then merged to rebuild the whole slice. Merged images were analyzed using ImageJ, and the percentage of area occupied by plaques was graphed.

### Cell culture

HT22 cells were seeded at a density of 1.25 × 10^5^ cells cm^−2^ in 21 cm^2^ culture dishes (ThermoFisher Scientific) in DMEM culture medium (ThermoFisher Scientific) containing 10% fetal bovine serum. Cells were grown to confluence in a 5% CO_2_ and 95% air humidified atmosphere. Cells were then incubated in DMEM without serum and exposed for 24 h to different concentrations of the Dyrk1 inhibitor (2.5–12.5 μm) dissolved in dimethyl sulfoxide (DMSO; 0.1% final concentration), or to the DMSO vehicle alone. To inhibit lysosomal activity, ammonium chloride was added at the same time of the Dyrk1 inhibitor (2 mm). Proteins were extracted in RIPA buffer and used for immunoblot assay.

### Antibodies

From Cell Signaling Technology (Danvers, MA, USA): phospho‐APP (T668) (Cat. #3823, 1:1000) and β‐actin (Cat. #3700, 1:10,000). From Millipore, Aβ42 (Cat. #AB5078P, 1:200), and Anti‐APP A4, clone 22C11 (Cat. #MAB348, 1:1000). From Covance (Princeton, NJ, USA), APP (Aβ amino acids 1–16) monoclonal antibody, 6E10 (Cat. #MAB1560, 1:3,000). From Abcam (Cambridge, MA, USA), Lamp2a (Cat. #ab18528,1:200) and phospho‐tau (S396) (Cat. #ab109390, 1:1000). From Sigma‐Aldrich (St. Louis, MO, USA), amyloid precursor protein (APP) C‐terminal (Cat. #A8717, 1:1,000). From ThermoFisher Scientific, HT7 (Cat. #MN1000, 1:5,000) and tau5 (Cat. #577801, 1:2,000). CP13 (1:1000) was a generous gift from Dr. Peter Davies.

### Statistical analyses

All data were analyzed using GraphPad Prism (GraphPad Software, CA, USA, www.graphpad.com). Data were analyzed by one‐ or two‐way ANOVA followed by Bonferroni's post hoc analysis or Tukey's multiple comparison tests, when applicable. Selective experiments were analyzed by Student's *t*‐test, as specified in the figure legends.

## Funding

This work was supported by grants to S.O. from the Arizona Alzheimer's Consortium and the National Institutes of Health (R01 AG037637).

## Conflict of interests

Christopher Hulme and Travis Dunckley have filed a provisional patent for the use of Dyrk‐inh as a treatment for Alzheimer's disease. The other authors have nothing to disclose.

## Authors contributions

CB designed the experiments, carried out the dosing of the mice, the biochemical and histological experiments, analyzed the data, and wrote the manuscript. DMS performed the behavioral experiments and contributed to the writing of the manuscript. RB performed some of the biochemical experiments and contributed to the writing of the manuscript. VG, AYS, CF, BS, CH, and TD developed the compound and contributed to the writing of the manuscript. AC analyzed the data and contributed to the writing of the manuscript. SO designed the study, analyzed the data, and wrote the manuscript.

## Supporting information


**Fig. S1** Markush structure of the Dyrk1‐inh.
**Fig. S2** Pharmacodynamic properties of Dyrk1‐inh.
**Fig. S3** Schematic representation of the treatment paradigm.
