# Supplementary material for: Dyrk1 inhibition improves Alzheimer's disease‐like pathology
Source: Aging Cell. 2017 Aug 4;16(5):1146–54. doi: 10.1111/acel.12648 (PMC5595697; doi:10.1111/acel.12648)
Supplement: Supplementary file 1 — Fig. S1 Markush structure of the Dyrk1‐inh. Fig. S2 Pharmacodynamic properties of Dyrk1‐inh. Fig. S3 Schematic representation of the treatment paradigm. [file ACEL-16-1146-s001.docx]

**
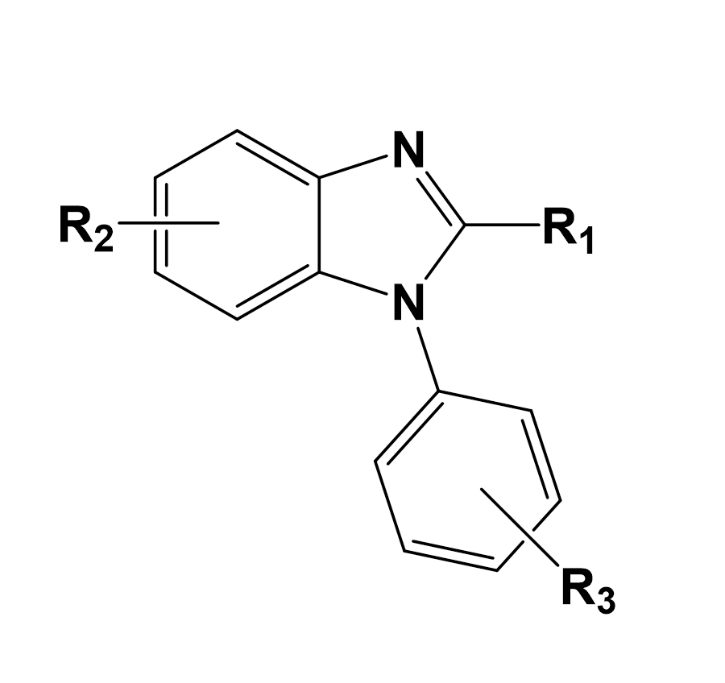
**

**Supplementary Figure 1. Markush structure of the Dyrk1-inh.**

The inhibitor was synthetized as detailed in Patent number US2016/050198.

**
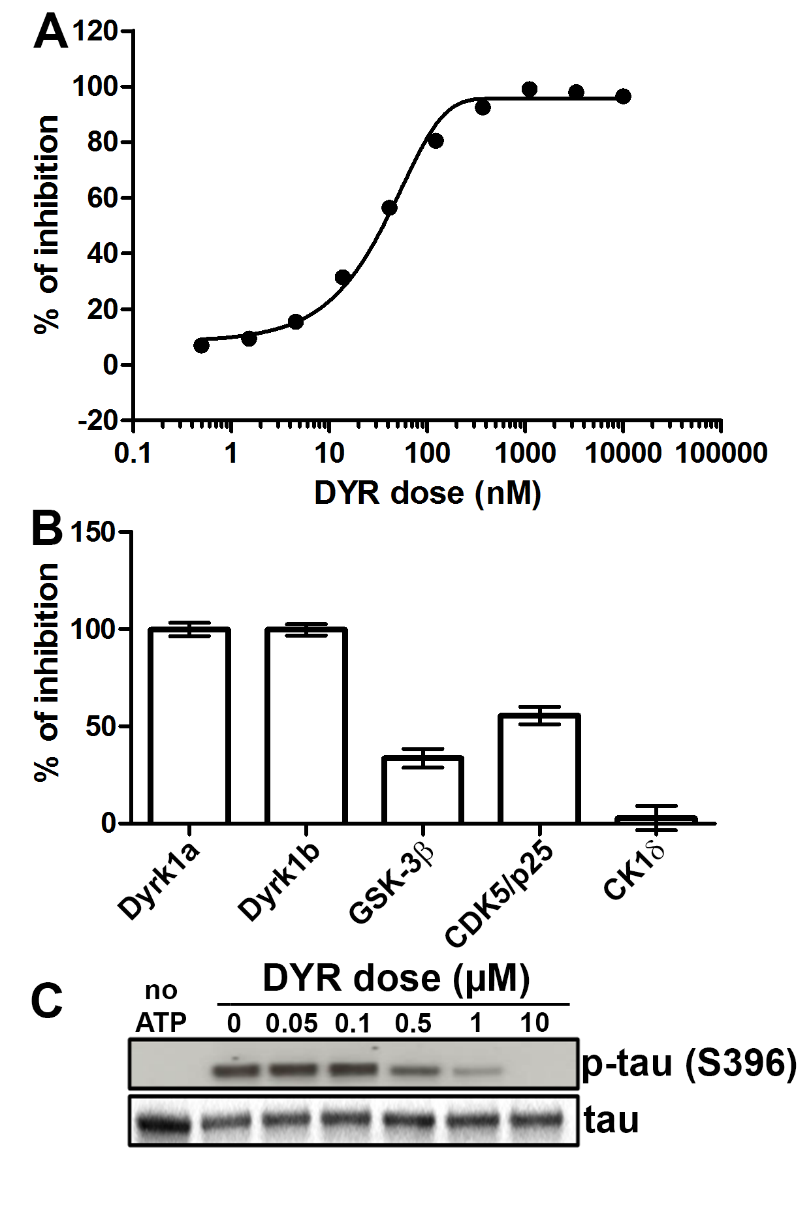
**

**Supplementary Figure 2. Pharmacodynamic properties of Dyrk1-inh**

**(A)** Inhibitory curve obtained with the Z'-LYTE™ Detection Kinase Assay Kit, performed using recombinant Dyrk1a protein and a fluorogenic substrate. **(B)** Percentage of inhibition on different kinases. Data were obtained by analyzing the inhibitory effect of 10 µM Dyrk1-inh with the EZ Reader Electrophoresis Mobility Chip Instrument. **(C)** Western blot analysis of recombinant tau, treated with increasing concentrations of the Dyrk1 inhibitor (DYR), in the presence of recombinant Dyrk1a. Dyrk1-inh reduced the phosphorylation of tau on S396, and this effect was dose-dependent.

**
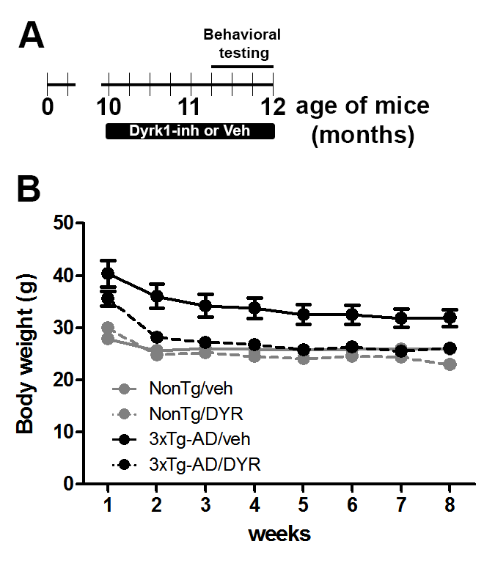
**

**Supplementary Figure 3. Schematic representation of the treatment paradigm.** **(A)** Ten-month-old female 3xTg-AD and NonTg mice were administered 12.5 mg/kg Dyrk1-inh or vehicle via daily intraperitoneal injections for 8 weeks (n = 13 for NonTg/veh, n = 15 for NonTg/DYR, n = 12 for 3xTg-AD/veh, and n = 13 for 3xTg-AD/DYR). During the last 3 weeks of injections, mice were tested in the open field and in the radial arm water maze (RAWM). **(B)** Weight change throughout the treatment. The 3xTg-AD/veh mice weighed significantly more than all the other groups during the whole treatment. Data are presented as means ± SEM.
